# Ectogenesis, gestational preferences and the social coercion argument

**DOI:** 10.1007/s40592-025-00253-2

**Published:** 2025-06-09

**Authors:** Jolie Zhou

**Affiliations:** Department of History and Philosophy of Science, University of Cambridge, Cambridge, UK

**Keywords:** Ectogenesis, Ectogestation, Adaptive Preferences, Coercion, Liberalism, Autonomy

## Abstract

This article challenges a subtle critique of ectogenesis—what I call the “social coercion argument” (SCA). The SCA holds that if ectogenesis becomes a standard gestational option, those who prefer pregnancy might be pressured into adopting it, thereby infringing on their autonomy and reinforcing inequality. On this view, ectogenesis might not be a morally sound solution to gender inequality. I first analyze the SCA within the liberal framework that underpins it. While its descriptive claim—that future women who prefer pregnancy may face pressure—may be valid, it cannot justify discounting the emancipatory potential of ectogenesis. I then examine some women’s preference for pregnancy over ectogenesis through feminist insights into adaptive preferences (APs). I argue that such preferences may be harmful and shaped by injustice, suggesting that gestational preferences are dynamic, and that addressing gender inequality requires strategies beyond cultural and social inclusivity. I conclude that the SCA’s core concern should be separated from the ethical evaluation of ectogenesis and addressed by continually “levelling up” choices.

## Introduction

The concept of artificial uteri is often referred to as “ectogenesis,” a term coined by British biologist Haldane (1933). In 2017, a research team in Philadelphia developed the “biobag,” a device that sustained extremely premature lambs for up to four weeks (Partridge et al. 2017). This breakthrough has sparked debate, including over terminology, as word choice may influence perceptions of the technology (Segers and Romanis 2022).

Notably, Kingma and Finn (2020) argue that the biobag is more accurately labelled as artificial amnion and placenta technology (AAPT), as it replicates the functions of the placenta and amniotic sac, not the womb itself. They propose using “ectogenesis” as a broad term for all mammalian development outside the maternal body, and “ectogestation” specifically for artificial gestation.

For the purpose of this article, I use “ectogenesis” to refer to the complete development of human offspring—from conception to birth—in an artificial (outside-the-body) environment, not limited to AAPT but inclusive of any enabling technology. This definition aligns with the article’s focus on women’s preferences between two modalities of having a child: pregnancy or full external development, encompassing both conception and full gestation. Conception refers to the formation of a zygote, while gestation is the process through which the conceptus develops into an entity with mature fetal physiology (Romanis 2022; 2024). This usage excludes cases where conception occurs in the body, but gestation is completed externally^1^, which are beyond this article’s scope.

Despite ectogenesis being regarded by some as a “pipe dream” (De Bie, Flake, and Feudtner 2023,558), Segers and Accoe (2023) note that speculation may signal ethical values that are already relevant today—such as those underscoring procreation. Indeed, since ectogenesis entered feminist discourse in the 1970s (Tong 2004; Segers 2021), discussions—particularly those concerning its emancipatory potential—have consistently revealed persistent gendered issues surrounding pregnancy. This article joins that strand.

Contemporary pro-ectogenesis scholars often build on Shulamith Firestone’s (2015) argument that gender oppression stems from reproductive differences between the sexes, contending that ectogenesis can enable fundamental gender equality if it becomes a safe and viable option (Singer and Wells 2006; Takala 2009; Kendal 2015). Some expand on this position by emphasizing ectogenesis’s potential to redistribute reproductive health burdens (Smajdor 2007, 2012) and to decouple women’s identity from pregnancy (MacKay 2020; Ferreira 2022). Others highlight its potential to support sexual and gender minorities in achieving reproductive equality (Kimberly, Sutter, and Quinn 2020; Segers 2021) and accommodate diverse gestational preferences (Bidoli 2025).

The opposing camp, while not strictly anti-ectogenesis, raises key concerns about its pro-equality potential:

(1) Gender oppression is primarily social rather than biological, meaning ectogenesis fails to address its root causes. It risks becoming “a red herring” (Cavaliere 2020, 80) or merely one component of a broader gender justice agenda (Cavaliere 2020; Campo-Engelstein 2020; Horner 2020; Romanis 2024).

(2) Stratified access to ectogenesis could undermine its emancipatory promise, as financial, legal, and cultural barriers may restrict who can benefit, thereby reinforcing existing inequalities (Romanis and Horn 2020; Horn and Romanis 2020; Cavaliere 2020; Horn 2022; Romanis and Kendal 2024).

(3) If ectogenesis becomes the standard mode of gestation, it may create social pressure against those who value and prefer pregnancy, potentially infringing on their reproductive autonomy.

This article focuses primarily on the *third* concern: the social coercion that operates “without requiring the force of law” (Horn 2022, 536). Some bioethicists are concerned that as ectogenesis becomes a safer option for the fetus, natural pregnancy may come to be perceived as inferior (Murphy 1989; Cavaliere 2020; Horn 2022; Romanis 2024). This perception could pressure individuals, especially those with less-than-ideal lifestyles or higher risk factors, to opt for ectogenesis (Jackson 2008; Horner 2020), leading to a loss of the pleasures derived from natural pregnancy (Murphy 2012). Cavaliere (2020, 79) argues that some women might still prefer pregnancy in the future, at which point ectogenesis “may end up undermining women who find meaning and self-fulfilment in gestation and childbirth. Their decisions to beget and birth children ‘naturally’ may be regarded as at odds with conceptions of the good life such as those advanced by proponents of ectogenesis, making opting out increasingly difficult. *Ectogenesis might hence not turn out to be equality-promoting*,” but instead exacerbate class divisions, further privileging some women over others.

This article defines this critique as the social coercion argument (SCA). Its core concern is that if ectogenesis becomes a safer option, it may become the norm, pressuring those who prefer pregnancy and infringing on their autonomy. The SCA thus questions whether ectogenesis is a morally sound means of promoting equality. It is worth noting that most SCA proponents do not see this concern as sufficient to reject ectogenesis outright but regard it as one of the reasons for discounting its moral potential to address gender oppression. Given its cautious and moderate stance, I term it an “argument” rather than a “rejection”.

This article first critically analyzes the SCA’s stance within the liberal framework and then further examines women’s preference for pregnancy from a feminist perspective. Accordingly, my argument is divided into two parts:

1. I argue that, while the SCA’s descriptive claim—that future women who prefer pregnancy over ectogenesis might face pressure—may be true, it does not constitute a valid reason to discount ectogenesis’s emancipatory potential within the liberal framework that underpins the SCA.
2. I examine gestational preferences—both current and future—through a feminist lens focusing on adaptive preferences (APs) that are harmful and shaped by injustice. Some women’s preference for pregnancy may constitute APs, implying that gestational preferences are dynamic and that addressing inequality requires strategies beyond cultural and social inclusivity.

Therefore, this article is structured as follows: Section 1 explains why concerns about future pressure on individuals who prefer pregnancy should not serve as grounds to discount the emancipatory potential of ectogenesis. Section 2 explores how some women’s preference for pregnancy over ectogenesis may constitute APs and examines the implications. Section 3 concludes that concerns about social coercion should be addressed independently, through the continued expansion of gestational choices.

## The SCA within liberalism

The SCA’s emphasis on diverse perspectives of the “good life” aligns with liberalism, which prioritizes respect for voluntary choice. Cavaliere (2020, 79), for instance, notes that support for ectogenesis reflects values typical of Western, Educated, Industrialized, Rich, and Democratic (WEIRD) societies—values that are not universally shared. Liberalism acknowledges societal pluralism, recognizing that individuals hold varied religious, moral, and personal beliefs about what gives life value (Levey 2005). Therefore, SCA authors argue that ectogenesis may impose a particular understanding of the “good life” on individuals, infringing on their autonomy.

What does this concern lead to? Some SCA proponents clarify that their stance is not to reject ectogenesis but to call for necessary social regulation or messaging to address this concern (Cavaliere 2020; Romanis 2020). Still, if achieving equality—the primary goal—through a technology or policy faces excessive barriers, its impetus weakens, prompting a search for alternatives. Therefore, SCA can be seen as a reason for *partially* rejecting ectogenesis. Here, I elaborate on a mild version of SCA:

1) There may be good reasons to develop ectogenesis to promote equality, particularly by accommodating diverse yet equally valid gestational preferences.

2) However, concerns remain that a new norm favoring ectogenesis might pressure those who prefer pregnancy into adopting it.

3) Therefore, without appropriate social regulation and messaging to prevent such pressure, ectogenesis’s emancipatory potential risks being diminished.

I broadly agree with the first two points but argue against using concerns about pressure from a new norm to assess the liberating potential of ectogenesis. I will first outline common ground before critiquing the SCA’s normative implications.

### Equal gestational preferences

People’s preference for ectogenesis, just like the preference for pregnancy, falls with the remit of procreative autonomy, at least within a liberal framework. Dworkin (1993) defines procreative autonomy as the right to control their own role in procreation unless the state has a compelling reason to deny it, akin to the right to freedom of expression. From this perspective, pursuing a safer way to have genetic children without undergoing pregnancy (including surrogacy) is not morally wrong. This preference may stem from concerns about career, health risks, child welfare, the social and legal implications of surrogacy, or a desire to decouple womanhood from pregnancy (Bidoli 2025). As Romanis and Kendal (2024, 113) note, reproductive choices are subjective and situated, shaped by personal values or intuitions. Even choosing ectogenesis to avoid stretch marks or bodily changes is not morally wrong in this framework, as it reflects beliefs about bodily integrity and aesthetics—just as valid as choosing pregnancy for its physical experience or identity-related meaning.

If gestational freedom should be somewhat restricted for a related third party, choosing riskier pregnancy becomes more questionable than opting for ectogenesis. For example, O’Neill (2002) argues that gestation aims to bring a third party—a child—into existence, often imposing care obligations that restrict rather than enhance autonomy. Similarly, Savulescu and Kahane (2009) assert that reproduction should be about having children with the best prospects. These views, which essentially suggest restrictions on riskier gestational choices (particularly for the fetus), are not without criticism. Still, even if these views do not plausibly challenge people’s preferences for riskier pregnancies (as compared to ectogenesis), we can at least say that, morally, preferring natural pregnancy and preferring ectogenesis are equally legitimate.

Yet, the reality is that the preference for ectogenesis remains unrealized. In a world without this option, women who desire genetic children but wish to avoid pregnancy or surrogacy face both social pressures—particularly pronatalist norms that define women as child bearers, caregivers, and mothers (Wilkinson and Williams 2016)—as well as technical and legal barriers. Realizing this preference would thus advance basic equality between individuals with differing gestational preferences.

### Potential social coercion

New norm pressure may emerge in the future, which, from a liberal perspective, indeed concerns individual reproductive autonomy. Liberalism is deeply committed to autonomy, affirming that “the individual is sovereign” (Mill 1989, 13) and that individuals must choose their way of life based on beliefs that give it value and meaning (Mill 1989,117). In general, several constraints might prevent people from making fully autonomous gestational choices. One is state and legal barriers; for instance, if ectogenesis were mandated as the only gestational option due to its superior health outcomes for babies, those who prefer pregnancy would be forced to comply. The second is resource-based coercion, where pregnancy remains formally permitted, but essential support is withheld, effectively pressuring individuals away from that choice.

The SCA, however, focuses specifically on a third type—social coercion. If ectogenesis becomes the dominant gestational choice and part of the standard life model, those who choose pregnancy might face stigma and discrimination. Indeed, even with strong anti-discrimination laws, subtle pressures from social norms—often exerted through informal, diffuse sanctions (Rodríguez López 2024) —may be difficult to avoid. For instance, those who opt for pregnancy might be subtly judged by society, partners, friends, family, and colleagues as selfish for not prioritizing their offspring’s health. It is therefore difficult to deny that, in the future, some individuals may feel pressured by the social endorsement of ectogenesis, making their gestational choices deviate from their preferences.

### Does the potential social coercion undermine the moral appeal of ectogenesis?

The key question is whether potential social coercion undermines the moral appeal of ectogenesis as a means of promoting equality. I argue that, from a liberal perspective, it does not.

Social coercion is a contextual issue—it arises not from ectogenesis itself, but from the possibility that societal norms might come to pressure individuals to choose ectogenesis—especially if it promises better fetal health—against their preferences.

Still, not all contextual consequences carry sufficient ethical significance to influence the moral evaluation of a development itself. For instance, the fact that a particular beer may be harmful to human health in certain contexts could indeed constitute an important ethical consideration regarding its production. In contrast, the popularity of a beer—potentially pressuring individuals who dislike beer to order it during social gatherings at pubs—is typically not considered an ethical factor relevant to decisions about its production. I now analyze why the concern about social coercion raised in the SCA lacks sufficient ethical significance to affect the ethical evaluation of ectogenesis.

First, the issue of social coercion raised in the SCA is extremely *universal*—it can apply to nearly any significant societal change, not just technological ones. Even initiatives widely considered progressive, such as promoting women’s education and workforce participation, might inadvertently generate norms that pressure women who prefer traditional homemaking roles into pursuing higher education or demanding careers. Similarly, advocacy for diverse spousal relationships that challenge heteronormative standards may, in the future, pressure those who prefer traditional marriages, making them feel marginalized. Despite these possibilities, scholars rarely assess the overall potential for progress of these changes based on such issues, and proponents of SCA are unlikely to do so either.

Second, the SCA highlights a *remote* risk for the current majority—one that would arise only if ectogenesis became widespread. Referring to such risks might subtly imply that expanding choices for current minorities should first account for potential future pressure on majorities aligned with existing norms.

Women who find meaning and fulfillment in pregnancy form the majority and reflect prevailing gender norms. In Western societies, the “perfect mother” ideal—prescribing (biological) motherhood as a central life goal and marker of womanhood—persists (Meeussen and Van Laar 2018). This does not mean these individuals are merely conformist; rather, it suggests that the current norm positions them as the standard and majority, while those who do not derive value from pregnancy are marginalized as deviants.

Women who most need or want ectogenesis for genetic children include those with uterine factor infertility and those who, despite being capable of gestating, choose not to—and reject surrogacy. These individuals deviate from pronatalist norms. In particular, the latter group remains largely invisible in public discourse and political agendas, with only limited recognition in academic discussions on ectogenesis. The development of reproductive technology also reflects a political agenda that overlooks their preferences, prioritizing innovations that enable pregnancy while neglecting gestational alternatives such as ectogenesis (Firestone 2015; Bidoli 2025).

We still live in a world where essentialist beliefs strongly link pregnancy and childbirth to the very definition of “womanhood” (Lotz 2018, 502). If debates over ectogenesis are intended to illuminate ongoing gender struggles (Cavaliere 2020), emphasizing a distant risk that individuals already aligned with prevailing gender norms might one day face pressure seems untimely and may convey some unintentional bias.

Third, and most significantly, the presumed harm in the SCA—autonomy infringement through social coercion—may *not* hold under a liberal conception of autonomy.

No choice is entirely free from social influence; even the most autonomous decisions are shaped by one’s background, upbringing, and experience, all contributing to one’s conception of the “good life.” Liberalism, as a political philosophy, seeks a just and legitimate framework for governance in a pluralistic society by recognizing diverse beliefs and ensuring fairness, stability, and mutual respect (Gardbaum 1996). This framework does not dismiss choices shaped by gendered norms but respects them as autonomous.

Some SCA supporters seem to uphold this autonomy standard when defending public funding for physical pregnancy technologies. For example, Cavaliere (2024) argues that even if gestational choices, such as uterine transplants, are influenced by patriarchal norms and might reinforce them, they should not be dismissed as mere false consciousness. In other words, even if societal norms pressure someone to see pregnancy as central to women’s identity and they undergo risky surgeries as a result, their choice is generally considered autonomous if it aligns with their own sense of well-being.

By the same standard, future cases of choosing ectogenesis under new normative pressures could still be considered autonomous. Human values and preferences are multifaceted—one can value the physical experience of pregnancy while prioritizing health for themselves and the fetus. The weight of these considerations is always shaped by external influences. As it is today, so it will be in the future.

When someone today chooses the risky uterine transplant to achieve pregnancy, their decision likely involves weighing multiple values and preferences. While they value their health, they may prioritize the identity and meaning attached to pregnancy, influenced by gender norms and societal expectations (Lotz 2018). Their choice reflects what they deem most important: health or the experience of pregnancy. In the future, someone might value the experience of pregnancy but prioritize fetal health (partly) due to societal norms that emphasize the latter. Under this pressure, they could opt for ectogenesis. If the former choice is considered autonomous, then so is the latter. Both are acting in accordance with their own multifaceted values, desires, and conceptions of the good life, balancing these according to their own sense of what is best. Their choices reflect their own interests, as they understand them (Romanis and Kendal 2024). From a liberal perspective, such choices are not necessarily non-autonomous.

Therefore, from a liberal perspective—which underpins the SCA—the concern about social coercion does not undermine ectogenesis’s liberating potential. In fact, this concern may be the weakest critique raised by critics. This is reflected in Romanis’s (2023) empirical study on the equality-enhancing potential of new assisted gestation: respondents primarily highlight barriers to access, with no concern about future social coercion against those who prefer pregnancy.

This is not to say that preferences for pregnancy over ectogenesis are unworthy of respect. On the contrary, stepping beyond liberalism into feminist critiques of how oppressive environments shape women’s preferences, the concern becomes even more profound. It shifts from simply respecting diverse gestational preferences to addressing deeper structural inequalities—an issue I explore in the next part.

## The SCA within the adaptive preference framework

SCA holders seem to project future preferences based on current attitudes toward pregnancy. It is true that some individuals today reject the prioritization of career advancement within Western values (Cavaliere 2020), enjoy being pregnant, and even campaign for the right to gestate and access womb transplants (Romanis et al. 2021). Following this trend, some future women may also prefer pregnancy. I generally agree with this possibility.

However, the way the SCA is framed lacks deeper inquiry into why individuals today—when imagining a safer alternative to pregnancy—and individuals in a future with ectogenesis might still prefer pregnancy. It is insufficient to attribute this solely to finding meaning, fulfillment, or identity through gestation. A more critical examination of the underlying causes of these preferences suggests that future gestational attitudes may be more dynamic. More importantly, scrutinizing the nature of these preferences could shift the focus from simply ensuring cultural or social inclusivity to addressing more substantive issues.

Some scholars have critically examined women’s preference for pregnancy over ectogenesis. Smajdor (2012) argues that women’s acceptance of gestational harm as pleasure may stem from the lack of alternatives and the normalization of gestational risks, making them less likely to question the value of pregnancy. Bidoli (2025) further suggests that the desire for pregnancy may reflect an adaptive preference shaped by oppressive constraints. However, no systematic analysis has yet been conducted within the AP framework—this section seeks to address that gap.

### What are adaptive preferences?

The concept of APs aligns with long-standing feminist concerns about the tension between feminism and liberalism or subjective welfarism. For example, Rawls (1999) argues that liberalism must allow traditional gendered divisions of labor in families if they arise from voluntary choices. However, women may “freely” prefer caregiving roles due to early gendered socialization. While liberalism protects such choices, they remain in tension with feminist critiques of gendered norms (Levey 2005). Subjective welfarism holds that individuals are the best judges of their own well-being. Yet, scholars such as Nussbaum (2000, 2011) and Sen (1984) argue that deprivation can impair people’s ability to evaluate their own welfare, explaining why some women may voluntarily endure abusive relationships or poor living conditions. Even collective preferences, such as heterosexual norms, may stem from indoctrination in male-dominated contexts (MacKinnon 1989). Simply fulfilling such preferences without scrutiny risks entrenching internalized oppression (Walker 1995) and reinforcing the patriarchal order (Cudd 2004).

A more systematic discussion is situated within the AP framework. Here, I outline several frequently discussed accounts of APs, including (1) Jon Elster’s procedural account, (2) Luc Bovens’ formal account, (3) Martha Nussbaum’s substantive account, and (4) Serene Khader’s perfectionist account, as summarized by Mitchell (2018). Through analysis, I endorse (3) and (4), contending that *harmfulness* and *injustice* are the two essential conditions for identifying APs.

Elster (1983) defines APs as those unintentionally formed towards feasible options, with their unintentional acquisition being the primary issue. He employs La Fontaine’s fable of the “sour grapes phenomenon” (SP) to illustrate this: when the fox cannot reach the grapes, she convinces herself that they are too sour and not worth desiring. Elster views this preference shift as irrational because it lacks deliberate character planning (CP) — a process of consciously fostering new preferences in response to constrained choices. If the fox had intentionally developed a preference for a reachable fruit after finding the grapes unattainable, the preference would be rational. Thus, the problem of SP, representing AP in Elster’s procedural account, is the absence of autonomous formation.

Bovens (1992) critiques Elster’s account and introduces the formal account. He argues that intentional acquisition cannot determine a preference’s rationality; an unintentionally formed preference can still be rational. For example, someone might enroll in a Spanish course to learn the language but unexpectedly develop a love for Spanish culture. Although this was not intentional, it is still rational. Instead, Bovens suggests “consistency” as a measure of rationality: a preference change is rational if it aligns with the holder’s other preferences. For instance, if the fox stops liking grapes but still enjoys grape-flavored food, that is inconsistent and irrational. If she shifts all her food preferences to non-grape-flavored fruits, the change is consistent and therefore rational.

Both Elster’s and Bovens’ accounts may offer some useful perspectives on why preferences for pregnancy over ectogenesis can be problematic. According to a small-scale survey, some women reject the idea of ectogenesis due to concerns that such machines could harm babies (Landau 2007). This may indicate an unintentional preference acquisition—they do not even envision that ectogenesis might better ensure fetal health. Additionally, a woman’s preference for pregnancy and acceptance of its harms may conflict with her lifestyle if she otherwise favors non-harmful means of achieving her goals, suggesting inconsistent preferences.

However, I reject procedural and formal accounts for examining women’s gestational preferences because they overlook the moral issues in the *content* of preferences and some of the *constraints* on choices. According to Elster, the problem arises only when individuals are unaware of these constraints; awareness supposedly resolves the issue. Similarly, Bovens identifies a problem only when a preference conflicts with the holder’s other inclinations; if consistent, it is morally unproblematic. Nevertheless, I argue that preferences, whether intentionally or consistently formed, remain morally concerning if they are harmful and shaped by unjust conditions.

Consider an alternative fable of the fox. Imagine the fox’s access to grapes is unjustly restricted because poachers drive her into an area with only poisonous berries. To survive, the fox decides to prefer these berries and dislike grapes. Through long-term deliberate effort, she even becomes addicted to similar harmful fruits. While Elster and Bovens might view this preference as intentional, consistent, and thus rational, it is clearly problematic. In other words, women may intentionally and consistently develop preferences to comply with oppression.

Footbinding^2^—a Chinese custom of binding young girls’ feet to keep them small (Ko 2005)—and female genital mutilation (FGM), involving partial or total removal of external female genitalia for non-medical reasons (World Health Organization 2025), are extreme examples of self-enforcing misogynistic practices. Women themselves often sustain and transmit these harmful traditions, feeling compelled to conform to societal expectations around marriage, sometimes resisting abolition efforts (Mackie 1996). Such preferences also match their daily prioritization of male interests. A more common contemporary example is women in abusive relationships preferring to stay, often because they weigh the consequences of leaving and internalize self-disadvantage, believing they deserve no better (Nussbaum 2000). These examples neither absolve perpetrators nor blame victims but illustrate that—even when preferences are deliberate and consistent—if they emerge from navigating oppression for immediate survival, they remain problematic and fall within the scope of APs.

The substantive and perfectionist accounts address issues of oppression more effectively, with the latter concretely articulating the former. APs are central to the capability approach (Nussbaum 2000, 2011; Sen 1984, 1987, 1990), which defines welfare through essential capabilities. Nussbaum (2011, 33) lists core capabilities like life, health, and bodily integrity. To thrive, individuals must engage with these; without them, true well-being is unattainable. Sen (1987, 45–46) describes:

A person who has had a life of misfortune, with very limited opportunities, and rather little hope, may be easily reconciled to deprivations than others reared in more fortunate and affluent circumstances. The metric of happiness may, therefore, distort the extent of deprivation, in a specific and biased way. The hopeless beggar, the precarious laborer or the dominated housewife, the hardened unemployed or the over-exhausted coolie may all take pleasure in small mercies and manage to suppress intense suffering for the necessity of continuing survival, but it would be ethically deeply mistaken to attach a correspondingly small value to their loss of well-being.

In other words, immediate benefits from compliance in an oppressive system cannot equate to the deprivation it enforces, nor does such satisfaction truly reflect well-being. I will explore this later.

It is worth nothing that for Nussbaum and Sen, most cases of APs are about local forms of injustice, such as those caused by deprivation in impoverished areas. Yet, there is no reason why they cannot also apply to cases of global injustice, where women worldwide are subjected to similar unjust conditions. Also, neither Nussbaum nor Sen provides clear standards for identifying APs, while Khader’s work addresses this gap. She clarifies key conditions for constituting APs^3^:

(1) They are inconsistent with an individual’s basic flourishing.

(2) They are formed under conditions not conducive to an individual’s basic flourishing.

(3) An individual can plausibly alter the preference into one that is more consistent with flourishing and endorse the change (Khader 2011).

I agree with Khader that conditions (1) and (2) are crucial for identifying APs. I simplify them as “harmfulness” and “injustice” respectively. While Khader’s formulation may be effective in other contexts, the simpler notions suffice for my argument on pregnancy and ectogenesis.

Harmfulness and injustice are both indispensable conditions. Preferences formed in response to injustice are not necessarily problematic. For example, a woman develops a preference for healthy eating and exercise to counter the “skinny” ideal imposed by misogynistic beauty standards. This is a resistant response. Though somewhat shaped by injustice, the preference benefits her well-being and is therefore not morally troubling. Conversely, preferences that involve some harm but are not shaped by injustice, such as a preference for risky sports, generally do not raise ethical concerns.

I cautiously set aside Condition (3)—that APs can be altered to align with fundamental goods—as it functions more as an ideal than a practical criterion. If preferences persist after intervention, we cannot know whether they were never APs or whether the intervention was simply ineffective. For example, if someone still prefers manual labor after AI replaces their job, either the preference reflects genuine enjoyment (and is not an AP), or it is an AP shaped by injustice, but AI replacement was the wrong intervention—they may need dignified, non-exploitative work instead. Thus, I define APs as preferences that are both harmful to the holder and formed in response to injustice. Within this framework, I examine whether some women’s preferences for pregnancy over ectogenesis constitute APs.

### The harm, trade-off, and injustice

Preferring pregnancy over ectogenesis prima facie meets condition (1)—it is harmful. Pregnancy imposes significant health risks on women, a fundamental aspect of well-being. Despite a global decline in maternal mortality over recent decades, a maternal death still occurred almost every two minutes in 2020 (World Health Organization 2020). Beyond pregnancies that carry significantly higher risks, even symptoms often considered normal—such as morning sickness, headaches, and bleeding gums—pose health risks higher than those of everyday life (Kendal 2015). Moreover, the impact of pregnancy extends beyond childbirth; about one in seven women may develop postpartum depression (Mughal, Siddiqui, and Azhar 2022); over a third of women experience lasting health issues postpartum (Vogel et al. 2024). Additionally, research shows that physical gestation adversely affects the gestator’s cognitive abilities due to gray matter volume (GMV) reductions during pregnancy (Pritschet et al. 2024), which persist postpartum, particularly in regions related to theory-of-mind processing—lasting for six years after parturition (Martínez-García et al. 2021) and detectable even decades later (Orchard et al. 2020). Thus, from the perspective of physical health, preferring physical pregnancy over ectogenesis is inevitably harmful.

However, a rebuttal—*the trade-off argument*—can be anticipated. One might argue that, despite the health risks, pregnancy could offer other crucial aspects of well-being that ectogenesis cannot, leading to greater overall good. Indeed, pregnancy-related well-being is multifaceted; if other significant benefits compensate for what is lost, the overall good may not be diminished. But the validity of this argument hinges on the specific contents of the trade-off—particularly whether the motivations behind it are shaped by injustice.

Considering concerns frequently raised by skeptics of ectogenesis, I examine three trade-offs: (1) maternal bonding, (2) personal experience, and (3) immediate interests within the system. I first outline why the first two—maternal bonding and personal experience—may not necessarily qualify as APs, and then focus on the third—immediate benefits within oppressive systems, which, I argue, exhibits characteristics of APs.

Maternal bonding and personal experience overlap to some extent and do not necessarily qualify as APs. In trade-offs, maternal bonding may refer to either a specific form of bonding or to a stronger degree of it—the former being closely tied to personal experience. The interaction between the fetus and the maternal body is rich; the fetus constantly touches the maternal body and perceives aspects of her social environment (Kingma and Finn 2020). Some women may prefer such unique intimacy, even if bonding does not necessarily diminish in the absence of pregnancy (Kennedy 2024). Personal experience may also involve viewing pregnancy as a meaningful challenge, akin to high-risk sports—difficult and potentially harmful, yet personally rewarding (Woollard 2020). Such preferences are not clearly shaped by injustice and can plausibly exist even in a gender-equal society. I therefore do not consider them APs.

The trade-off for “better” bonding is likely more complex. The assumption that pregnancy fosters a stronger bond is questionable—many pregnant individuals do not experience prenatal bonding, and assuming pregnancy inherently creates unique parental attachment overlooks individual variation (Romanis 2024, 149). Studies show that oxytocin—the “love hormone”—is released at similar levels in biological and non-biological parents during sustained caregiver-infant interaction, especially through skin-to-skin contact (Cong et al. 2015). This suggests bonding via ectogenesis may not be diminished with adequate care. What matters here is the belief itself that ectogenesis—the “cold machine”—undermines maternal bonding (Landau 2007). This concern may reflect fear of new technology or uncertainty, not necessarily injustice. Even in a fully gender-equal society, similar views might persist due to limited evidence. However, when such beliefs are reinforced by social norms tying womanhood to unconditional maternal love and pregnancy, they begin to intersect with the third trade-off, which I address below.

More scientific research and ethical debate are needed regarding “bonding” and “self-experience.” I particularly focus on the third type of trade-off—using physical pregnancy to seek *immediate benefits* within oppressive systems, as emphasized by Sen (1987). This may be the most entrenched reason for valuing pregnancy in pronatalist societies, leading some to dismiss ectogenesis for fear of losing something important, often without critical reflection.

Consider the warning from Robyn Rowland: since “for the history of mankind, women have been valued primarily as child-bearers,” the advent of ectogenesis might leave women “without a product of any kind with which to bargain,” potentially rendering them obsolete (Rowland 1987, 45).

However, rejecting ectogenesis due to fears of diminishing women’s status as primary child-bearers is defeatist. Such a rejection mistakenly assumes that women’s social progress relies on negotiating with patriarchy through pregnancy. It also exaggerates the significance of exclusive biological capabilities for achieving equality. If exclusive biological functions were essential for overcoming oppression, then anti-racism and anti-classism efforts would be inherently futile—since these marginalized groups lack unique biological capacities compared to dominant groups.

Nonetheless, Rowland’s warning accurately exposes a deeper societal phenomenon: in patriarchal systems, women’s identity and value have historically been tightly tied to their reproductive role. This may have fostered widespread anxiety and fear that women could lose the “social significance” assigned to them by oppressive structures if their gestational roles become nonessential.

This fear illuminates a critical mechanism behind trade-off (3): the excessive binding of women’s identity and value to pregnancy. Such binding typically results from systematically curtailing women’s opportunities to find fulfillment beyond childbearing and rearing. Consequently, many women internalize this association, actively pursuing the identity, recognition, and self-worth tied exclusively to pregnancy, thus developing a preference for pregnancy over ectogenesis.

The connection between women’s identity and their roles in childbearing is not inherently problematic, as individuals have the right to integrate their biological capacities into their identity. Rather, the issue lies in degree: when women’s opportunities for achieving identity and self-worth beyond reproduction are systematically limited, they are subtly coerced into seeking recognition primarily within the reproductive domain—the narrow space permitted by patriarchy—thereby rendering this connection excessive. Such an excessive degree of binding is unjust. A just response to women’s contributions to human reproduction should not involve restricting or disciplining them into prioritizing pregnancy; instead, it should entail greater investment in empowering women to realize their value beyond reproduction (Zhou 2025a).

However, even in today’s Western societies—where women can largely gain identity and value through careers and personal interests—the link between women’s reproductive function, the identity category of “woman,” and the social role of “mother” remains deeply entrenched (MacKay 2020). Infertile women’s social identity can still be severely damaged in these societies (Alamin et al. 2020). This is even more pronounced in more overtly oppressive contexts, where refusing or being unable to conceive can be prohibitively costly. For instance, studies on infertility among Nigerian women reveal that infertility often leads to abusive marriages, divorce, poor living conditions, abandonment, and mistreatment in old age (Larsen et al. 2010).

When opportunities for fulfillment beyond reproduction are systematically constrained—and reinforced by social discipline and sanction—women as a group may internalize these imposed values, coming to view pregnancy as central to their identity and deriving self-worth from it. This creates a cycle: shaped by gender norms from an early age, women’s beliefs and interests revolve around reproduction, making them more inclined toward caregiving and housework. Fulfillment from these roles reinforces the belief that childbearing defines their identity, further entrenching pronatalist norms and gestational preferences. This tight binding of identity to pregnancy ensures women’s cooperation in sustaining patriarchy, as Marilyn Frye notes: “efficient exploitation requires that those exploited be relatively mobile, self-animating, and self-maintaining” (Frye 1983, 59).

Another key mechanism underpinning the trade-off 3 is probably the collective *normalising* of gestational harms and risks. Historically, childbirth pain has been deemed natural and even meaningful, a belief reflected in religious doctrines that have hindered the development and application of pain relief in childbirth (Pence 2006). Childbirth trauma was once dismissed as a sign of women’s psychological weakness (Michaels 2018). Today, one in 20 mothers in the UK develops post-traumatic stress disorder (PTSD) due to unbearably painful childbirth experiences (Triggle 2024), which is closely linked to obstetric violence (Silva-Fernandez et al. 2023)—manifesting as “negligent, reckless, discriminatory, and disrespectful acts by health professionals, legitimized by power structures that normalize and trivialize their occurrence” (Jardim and Modena 2018, 7). Nearly half of mothers with postpartum depression go undiagnosed, fearing abandonment and lack of support (Beck 2006). Despite widespread suffering from long-term postpartum conditions, these issues remain historically neglected (Vogel et al. 2024).

Even severe gestational harm and risk are treated as signs of maternal suitability and often go undertreated due to their perceived normality (Smajdor and Räsänen 2025). At the same time, women face excessive medicalization aimed at minimizing fetal risks, such as unnecessary C-sections (Romanis et al. 2021). Though seemingly contradictory, these practices are unified in their harm to pregnant individuals (Zhou 2025b) and align seamlessly with the “normalcy” narrative: because common gestational harms are viewed as natural sacrifices, addressing them is seen as unnecessary, while further sacrifices for fetal well-being are framed as justified.

Very likely, the collective marginalization of gestational pain and suffering has led women to view it as taken for granted and even essential for good motherhood. Consequently, they may see enduring this pain for social recognition as a worthwhile trade-off rather than considering other options.

Both mechanisms behind trade-off 3—fostering women’s identity and value around pregnancy and idealizing gestational pain and harm as a necessity of good motherhood—may have influenced women’s preference for pregnancy. These mechanisms are deeply unjust, and even typical pregnancies can be harmful to women’s health overall; therefore, preferences for pregnancy driven by this third trade-off are APs.

### Implications

I have argued that, so far, some women’s gestational preferences may be APs. I contend that this suggests two key points. The first is *predictive*: with the emergence of ectogenesis and related societal changes, women’s gestational preferences are likely to shift significantly, while some APs may persist. The second is *normative*: the current preference for pregnancy over ectogenesis does not reliably reflect women’s well-being, and future APs may necessitate societal efforts to rectify relevant injustices. Given that the exact trajectory of technological development and societal response cannot be precisely predicted (Schick 2016), I will only briefly discuss two implications in a general and principled manner.

#### The future dynamics of gestational preferences

Paradoxically, history seems to suggest that women, who bear gestational harms and risks, are less enthusiastic about ectogenesis than men. As mentioned earlier, the term “ectogenesis” was coined by Haldane (1933), a male biologist. Aldous Huxley’s Brave New World (2004) popularized the literary image of artificial wombs.

Nevertheless, with the rise of the feminist movement, women’s desire for ectogenesis has increasingly been voiced. Firestone (2015) has argued that ectogenesis, accompanied by other revolutionary changes, is essential for women’s liberation—a view later echoed in Marge Piercy’s feminist novel Woman on the Edge of Time (1976). Western middle-class women have been the primary consumers of both the discourse and practice of artificial reproduction, surrogacy, and ectogenesis research (Aristarkhova 2005, 53). This trend is likely not coincidental. In societies with greater gender equality, women are less reliant on patriarchal incentives for childbearing. Women who prioritize roles beyond childbearing are less likely to tie their identity to pregnancy and more open to acknowledging its harms and risks. As patriarchal structure diminishes and gestational choices expand, many women may favor ectogenesis, though it is likely not a straightforward process.

Shifts in attitudes toward formula feeding might offer clues for prediction. Initially popular among upper-class families, formula feeding later became stigmatized as the benefits of breastfeeding were overstated (Buturovic 2020). If history repeats itself, ectogenesis may provoke a similar backlash, with higher classes first embracing its exclusivity, and abandon it as natural pregnancy regains prestige. More specifically, ectogenesis (once proven safe) may first be a luxury accessible only to the privileged, while underprivileged groups lack access. Over time, this dynamic could reverse—natural pregnancy, requiring extensive medical and social support, might become a status symbol among the elite, valued for its experiential aspect and exclusivity, while wage-dependent individuals may turn to ectogenesis to ease pregnancy burdens and enhance career and economic stability.

But eventually, if stronger evidence confirms ectogenesis’s superior safety for babies, it is likely to become the dominant gestational choice, as critics predict. If this happens in the future, it may indicate that the previous preference for pregnancy was largely shaped by a lack of choice and a gendered culture. Still, some may prefer pregnancy for the experience itself, while others may do so due to limited access to ectogenesis or the influence of unjust cultural factors. There can be two general scenarios:

Scenario 1 is that some women may lack substantial access to ectogenesis due to *unequal resource distribution*. Especially in its early stages, ectogenesis is expected to be highly expensive (Romanis and Kendal 2024, 149). While it may become a norm-like choice for middle- and upper-class individuals, it could remain inaccessible to women in impoverished communities. For these women, the reality of deprivation may lead to a form of APs—where, as Nussbaum (2000, 68) notes, “it becomes less painful to be kept in total neglect; otherwise, she is bound to suffer, and suffer pointlessly, the pain of injustice, if she cannot change the rules governing her life.”

As I introduced at the beginning, stratified access to ectogenesis is another major concern raised by SCA holders. However, this concern has not been directly linked to the formation of gestational preferences. Once we make this connection, it suggests that disparities in access to ectogenesis may not only determine who can utilize the technology but also shape the collective psychology of deprived communities. This means that those lacking substantial access to ectogenesis would not only face the pressure of a new societal norm but also the deprivation itself.

Scenario 2 could arise from familial and community traditions. If one’s religious community or family upholds traditional beliefs that tie women’s worth to gestational capabilities and glorify the pain and suffering of childbirth, this may still foster APs for pregnancy. For example, despite legal and social bans on practices like the above-mentioned FGM and “honor killings”—the murder of women and girls by male relatives for perceived sexual misconduct—some migrant communities in Western countries, such as the UK, continue these practices to maintain their cultural identity (Njue et al. 2019; Doğan 2013). These harmful practices illustrate how cultural communities can deviate from mainstream norms to uphold their traditions. Thus, even in a broader society that prioritizes ectogenesis, certain communities or families may impose strict prohibitions against it, with members facing condemnation or punishment for using it.

Both scenarios meet the two basic conditions of APs: harmfulness and injustice. Deprivation and certain community or family cultures that devalue women may continue to foster APs for pregnancy. In both cases, AP holders endure *double* harm—oppression stemming from deprivation and unjust traditions within their communities, while misunderstanding or discrimination from the broader society that advocates ectogenesis further marginalizes and pressures them.

#### Normative implications

Khadar (2011), Sen (1987), and Nussbaum (2011) suggest that the presence of APs indicates preferences might not accurately reflect individuals’ well-being, and thus, should not be the primary basis for policymaking. I generally agree with this view.

It is likely that a large proportion of women today may not support ectogenesis. However, given the presence of APs, such preferences should not be taken as direct or definitive evidence for informing relevant policies. This is not to undermine democratic principles; rather, it underscores the need for extensive interdisciplinary public dialogue, including ethical debates on oppression and the shaping of preferences.

Regarding potential future APs for pregnancy, I contend two overarching principles. On the one hand, women’s legitimate rights to pregnancy must be upheld regardless of the AP phenomenon. One compelling reason is that bodily autonomy grants women the right to decide what happens to their own bodies (Thomson 1976), thereby protecting pregnancy rights from any coercive interference. Although bodily autonomy is often invoked in defense of abortion rights, it also inherently supports the right to choose pregnancy. Further discussion of this principle is unnecessary here, as the SCA does not involve scenarios where pregnancy is legally prohibited.

On the other hand, while I oppose paternalistic interference in gestational choices, I argue that it is crucial to examine the unjust factors that may influence these preferences. If some women prefer or choose pregnancy due to a lack of access to ectogenesis or because of oppressive familial or community environments, this does generate a societal responsibility to address and rectify these conditions.

How to intervene? Surely, victims of double harm from both their immediate surroundings and broader society should not be blamed or coerced into choosing ectogenesis. Instead, efforts should empower women to choose their gestational methods free from injustice. On this point, I am not fundamentally at odds with the SCA proponents, as I support calls for greater societal action.

However, my approach may diverge somewhat from what the SCA framework implicitly suggests. Within the SCA, addressing social coercion logically appears to involve creating an inclusive cultural environment to accommodate diverse preferences. Yet, when linked to APs, this approach is clearly insufficient and may even obscure deeper issues. It is akin to advocating for tolerance of someone living an extremely minimalist lifestyle without addressing the underlying causes, such as poverty or past trauma. Such an approach may fail to resolve the actual problem. Similarly, promoting inclusivity alone, without confronting the unjust forces that shape gestational preferences, risks repeating a similar oversight.

Perhaps the concern raised in the SCA, and issues related to limited access to ectogenesis could be addressed together to some extent. In cases where unequal resource distribution restricts access to ectogenesis, state-sponsored programs (Kendal 2015) or other public funding initiatives could help. Where harmful community or family norms shape preferences, lessons from how liberal states address similar issues may be instructive, though each case requires separate consideration. Since proposing specific solutions based on rough predictions of future complexities is imprudent, I refrain from further elaboration here. The key point is this: only when the injustices shaping a preference for pregnancy over ectogenesis are removed will gestational preferences more accurately reflect individual well-being and calls for an inclusive culture be better positioned to alleviate related forms of pressure.

## Reconciliation: leveling up choices

The SCA warns of a distant scenario in which ectogenesis becomes dominant, pressuring those who find meaning in pregnancy to conform. Combined with other concerns, it questions whether ectogenesis is a morally sound solution to gender oppression. While some SCA advocates frame them as a call for regulation and social messaging, the underlying logic implies that if these issues are difficult to resolve or risk reinforcing inequality, ectogenesis should be dismissed.

I have argued that the potential for social coercion does not undermine the moral desirability of ectogenesis as a pro-equality tool within a liberal framework. Such issues are overly universal—even widely recognized progressive social changes can generate social coercion for those who prefer previous ways of life. Judging the expansion of choices for the current minority based on the distant risk to the current majority inadvertently conveys an unpromising attitude. More importantly, from a liberal perspective—which maintains a high tolerance for the influence of gender norms on people’s preferences and values—future choices for ectogenesis, even under social pressure, can still be autonomous.

This is not to say that respecting the preference for pregnancy is unimportant. Rather, I suggest scrutinizing gestational preferences through a feminist lens, particularly considering internalized oppression. Why do women often prefer pregnancy over ectogenesis, even if the latter is safer? It is insufficient to assume this preference stems purely from joy, experience, or bonding. While women may genuinely prefer pregnancy, gendered norms have long shaped—and will continue to pressure—women into viewing it as central to their worth. Internalized oppression blurs the line between personal preference and cultural conditioning, making it difficult to determine whether such choices are truly autonomous or socially ingrained.

If ectogenesis eventually becomes the standard gestational choice, this shift would itself suggest that women, as a group, have taken the opportunity to critically “reevaluate certain assumptions regarding women’s bodies and their roles in procreation, which have historically been taken for granted” (Kendal 2015, 70). For those who continue to prefer physical pregnancy in the future, their preference will likely fall into two broad categories: some shaped by deprivation or unjust community and family norms, and others motivated by a desire for meaningful experiences. Both deserve protection, but through different approaches. For those affected by deprivation or unjust cultural expectations, their preference should be understood as a signal to address deeper injustices. Only when women are fully informed about both gestational options and have substantial access to both can their choice be considered truly autonomous. At that point, cultural inclusivity will be more effective in eliminating stigma and discrimination against pregnant individuals, rather than obscuring deeper issues.

Regardless of how difficult it may be to address these concerns, they should never justify rejecting ectogenesis, even partially. The morally appropriate solution lies in expanding substantive gestational choices. While “leveling down” by restricting options to prevent inequality may seem appealing, it ultimately deprives everyone of meaningful choices and exacerbates the problem. Instead, “leveling up” by expanding options empowers individuals without limiting others’ freedom (Reaume 2013). This approach is essential when addressing constraints on gestational preferences, whether due to material deprivation or cultural pressures.

In general, the potential social coercion raised in the SCA should be addressed separately from the ethical evaluation of ectogenesis. The appropriate response to such concerns should focus on expanding gestational choices—both materially and culturally—rather than leveling down existing or potential options.

## Data Availability

No datasets were generated or analysed during the current study.

## Footnotes

1 For example, Mangione (2025) describes reciprocal effortless IVF, which involves a device that mixes an egg with Anna Smajdor for suggesting into an individual’s vagina or uterus for several hours or days to support fertilization and early embryonic development. The resulting entity is then removed, cryopreserved, and implanted into another individual. In theory, it could also be transferred to an external artificial device (once available) for full gestation. This article does not address such cases, but instead focuses on individuals’ preferences between full pregnancy (regardless of fertilization method) and the complete external development of human offspring, including external fertilization.

2 Thanks to Anna Smajdor for suggesting this example in a conversation.

3 Khader uses the term “inappropriate adaptive preference” to suggest that adapting to limited choices is not inherently wrong. However, I find “inappropriate” unnecessary because none of the aforementioned accounts view “adaptive” as merely “adapting.” In other words, “adaptive preferences” in their accounts refer exclusively to problematic ones, even though they may have different standards for determining what is problematic.
